# CONCURRENT VALIDITY OF THE BRUNET-LÉZINE SCALE WITH THE BAYLEY SCALE FOR ASSESSMENT OF THE DEVELOPMENT OF PRETERM INFANTS UP TO TWO YEARS

**DOI:** 10.1590/1984-0462/;2017;35;2;00005

**Published:** 2017

**Authors:** Fernanda Guimarães Campos Cardoso, Cibelle Kayenne Martins Roberto Formiga, Thailyne Bizinotto, Rogério Blasbalg Tessler, Francisco Rosa

**Affiliations:** a Universidade do Estado de Santa Catarina, Florianópolis, SC, Brasil.; b Universidade Estadual de Goiás, Goiânia, GO, Brasil.

**Keywords:** psychometrics, child development, validity of tests

## Abstract

**Objective:**

To verify the correlation between the areas evaluated by the Brunet-Lézine and the Bayley III scales of preterm infants up to two years.

**Methods:**

The study included 88 children who were divided into 3 groups: Group 1 (1 month to 5 months and 29 days of corrected chronological ages - CCA) with 32 children; Group 2 (6 months to 11 months and 29 days of CCA) with 36 participants; and Group 3 (18 -23 months and 23 days of CCA) with 20 children. The concurrent validity of the Brunet-Lézine scale and the Bayley III scale was calculated using the Pearson correlation or its non-parametric version, the Spearman correlation.

**Results:**

Group 1 showed moderate correlation between the developmental quotient for hand-eye and fine motor coordination (DQE), and fine motor score (ρ=0.448; *p*=0.01). Group 2 had moderate correlation between the developmental quotient for posture and gross motor function (DQP), and the gross motor score (ρ=0.484; *p*=0.003, between the DQE and fine motor score (r=0.489; *p*=0.002), and between the developmental quotient for social reactions (DQS) and the socio emotional score (r=0.435; *p*=0.008). Group 3 showed moderate correlation between the DQP and the gross motor score (ρ=0.468; *p*=0.037) and strong correlation between developmental quotient for language (DQL) and the score of language (r=0.890; *p*<0.001).

**Conclusions:**

The Brunet-Lézine scale showed strong correlation with the Bayley III scale regarding the language domain in Group 3, suggesting its validity to assess the language of children aged between 18 and 24 months.

## RESUMO

**Objetivo:**

Verificar a correlação entre as áreas avaliadas pela Escala Brunet-Lézine e pela Escala Bayley III no desenvolvimento de bebês pré-termo de risco até dois anos de idade.

**Métodos:**

Foram incluídas no estudo 88 crianças, divididas em 3 grupos: Grupo 1 (1 mês a 5 meses e 29 dias de idade cronológica corrigida - ICC), com 32 crianças; Grupo 2 (6 meses a 11 meses e 29 dias de ICC), com 36 participantes; e Grupo 3 (18 meses a 23 meses e 23 dias de ICC), com 20 crianças. A validade concorrente entre a Escala Brunet-Lézine e a Escala Bayley III foi calculada por meio da correlação de Pearson ou de sua versão não paramétrica, a correlação de Spearman.

**Resultados:**

No Grupo 1 houve correlação moderada entre o quociente de desenvolvimento da coordenação óculo-motriz (QDC) e o escore motor fino (ρ=0,448; *p*=0,01). No Grupo 2 foi encontrada correlação moderada entre o quociente de desenvolvimento da postura (QDP) e o escore motor grosseiro (ρ=0,484; *p*=0,003), entre o QDC e o escore motor fino (r=0,489; *p*=0,002) e entre o quociente de desenvolvimento de sociabilidade (QDS) e o escore socioemocional (r=0,435; *p*=0,008). No Grupo 3 foi observada correlação moderada entre o QDP e o escore motor grosseiro (ρ=0,468; *p*=0,037), e correlação forte entre o quociente de desenvolvimento da linguagem (QDL) e o escore da linguagem (r=0,890; *p*<0,001).

**Conclusões:**

A Escala Brunet-Lézine apresentou forte correlação com a Escala Bayley III quanto à linguagem no Grupo 3, o que sugere a sua validade para avaliar crianças entre 18 e 24 meses de idade nesse quesito.

## INTRODUCTION

The use of validated instruments is essential to establish a standardized language between professionals of different areas. They allow the comparison of data throughout time and the conference of therapeutic techniques and approaches, besides providing scientific base to understand and analyze the problems observed. The process of validation assesses the correction and relevance of a proposed interpretation, that is, it evaluates what is measured by the test, and how well it measures the data that need assessment. Validation can be subdivided into criterion validity, content validity, and construct validity.^1^ ^,^ ^2^ ^,^ ^3^ ^,^ ^4^ ^,^ ^5^ ^,^ ^6^ ^,^ ^7^

Concurrent validity represents the relation of the scores in the analyzed test with the scores of another test, preferably a test considered as “gold standard”, in the same construct. By comparing the results of the test with the reference ones, a measure is obtained to function as a diagnostic reference. This type of validity is especially interesting for physical therapists in order to decide which instrument to use in their practice.^3^ ^,^ ^4^ ^,^ ^5^ ^,^ ^7^ ^,^ ^8^ ^,^ ^9^Predictive validity examines the accuracy of the scores of an evaluation instrument, administered in a specific period, to predict future outcomes in the child’s development.^3^ ^,^ ^5^

The Scale of Psychomotor Development of Children, also known as the Brunet-Lézine Scale, was validated for the French population,^10^ but there is a modified version used in studies conducted with the Brazilian children present no delays in psychomotor development.^11^ Among the benefits of using this scale are the easy administration, the short time of application, and the low cost of training and acquisition of materials. These qualities, when associated with good psychometric properties, are essential to choose the instrument and favor its use in follow-up of outpatient clinics*.* ^4^ ^,^ ^5^ ^,^ ^12^

On the other hand, one of the most used assessment instruments of development for children all over the world is the Bayley Scales, applied to measure the cognitive development of preschoolers. Its second version is mostly used to identify children with developmental delay;^8^ ^,^ ^13^ ^,^ ^14^ ^,^ ^15^ ^,^ ^16^ ^,^ ^17^ ^,^ ^18^The Bayley Scales are considered the gold-standard to assess childhood development; therefore, they are commonly used in the world, in comparison to other instruments of psychomotor development evaluation.

In this context, the objective of this study was to verify the correlation between the areas assessed by the Brunet-Lézine and the Bayley Scale III in the development of preterm infants at risk up to the age of two years.

## METHOD

This study included 88 children, 45 male (51.1%) and 43 female (48.9%), assessed between January and October 2011. The following inclusion criteria were adopted: birth weight <1500 g and attending the High Risk-Infant Outpatient Clinic at the University Hospital Polydoro Ernani de São Thiago, in Universidade Federal de Santa Catarina (UFSC), or the High-Risk Follow-up Clinic Carmel Dutra, during the period of data collection. Exclusion criteria were diagnosis of genetic conditions, and congenital and/or heart malformation.

The following instruments were used for assessment: the Bayley III Scale and the Scale of Psychomotor Development of Children (the Brunet-Lézine Scale), in a single opportunity. The order of application was random. The scales were administered by two researchers, each of whom was in charge of one of the instruments from the beginning to the end of the study. Both researchers were trained for both instruments, and the reliability rate obtained for the application was considered adequate.

The Bayley Scale was created by the American psychologist Nancy Bayley, in 1969, and revised in 2006. It is composed of three sub-scales carried out by the child: cognitive scale, language scale (receptive and expressive communication), and motor scale (fine and gross motor areas). It is also composed of the socioemotional scale and the adaptive behavior questionnaire, which is answered by the people in charge of the child.^21^

The Scale of Psychomotor Development of Children (the Brunet-Lézine Scale) is a French scale, developed in the 1950s by Odette Brunet and Irène Lézine. After going through some changes, it was published in 1976. The manual and the items were translated to Portuguese in 1981.^10^ The scale aims at assessing children aged between 1 and 30 months as to the following areas of development: posture, hand-eye coordination, language, and sociability.

Considering the corrected chronological age (CCA) at the time of evaluation, the sample was divided into three groups: Group 1 (1 to 5 months and 29 days of CCA), with 32 children; Group 2 (6 to 11 months and 29 days of CCA) with 36 children; and Group 3 (18 to 23 months and 23 days of CCA) with 20 children. Prematurity correction was carried out by subtracting the chronological age according to the time left to complete 40 weeks.

Considering that the Brunet-Lézine Scale is conducted month by month, in Group 1 the choice was to include only children aged more than one month of CCA; this decision aimed at preventing children aged less than a month old did not perform any item, thereby generating zero score, this would lead to the underestimation of the child’s performance.

When the children showed mild developmental delay, their tutors were advised as to the proper stimulation. Children with severe developmental delay were referred to specialized professionals after communication with the doctor in charge of the follow-up.

The calculation of concurrent validity between the Brunet-Lézine and the Bayley III scales was conducted based on the correlation between the scores in the areas assessed by the Brunet-Lézine score in relation to the scores in the Bayley III Scale, after the latter was corrected by constant -7. The scale manual found means seven points higher than the means found by the Bayley Scale evaluation.^21^ To neutralize the overestimation of development, a “correction” was carried out in the scores of the Bayley III scale, subtracting seven points from all composed or converted scores, aiming at analyzing, in a more reliable way, the psychomotor development, and to improve the precision of delay diagnoses.

Gross motor skills were analyzed based on the correlation between the developmental quotient for posture (DQP), in the Brunet-Lézine Scale and the gross motor score in the Bayley III Scale. The fine motor skills were related to the correlation between the developmental quotient for hand-eye coordination, in the Brunet-Lézine Scale, and the fine motor skills in the Bayley III scale. In the language field, there was a correlation between the developmental quotient for language (DQL) in the Brunet-Lézine scale, and the score of language in the Bayley III scale. To assess the sociability factor, the quotient for social reactions (QSR), in the Brunet-Lézine Scale, was correlated with the socioemotional score in the Bayley III Scale.

The concurrent validity between the Brunet-Lézine and the Bayley III Scales was calculated using the Pearson correlation, or its non-parametric version, the Spearman correlation. The levels of correlation were categorized - based on the following classification: 0.00-0.19, weak; 0.20-0.39, very weak; 0.40-0.59, moderate; 0.60-0.79, strong; and >0.80, very strong.^22^ The analysis of sensitivity, specificity, positive and negative predictive values, and accuracy of the Brunet-Lézine Scale was conducted by the relations between the delay diagnoses in this scale with the Bayley III Scale; the values were corrected by the -7 constant. These measures were calculated based on the results of all participants. Data were exposed in the matrix for calculation. This demonstration in the matrix and the precision measures of the Brunet-Lézine Scale were obtained using formulas in the literature about clinical epidemiology.^23^ The use of scales that preferably have sensitivity values higher than 80% is recommended, as well as specificity higher than 90%, and positive predictive values higher than 70%.^4^ Therefore, this study classified values above this recommendation as adequate.

## RESULTS

In this study, the biological data had the following results, described as mean±standard deviation: birth weight 1140±236 g; birth height 36.9±2.8 cm; and gestational age (GA) 212±14 days. There was no statistical difference between the means of birth weight and GA in the three groups, according to the test of analysis of variance - ANOVA (F=0.303 and *p*=0.739 for birth weight; and F=0.697 and *p*=0.501 for GA).

The performance of children in Group 1, in both scales, and the correlation between the developmental areas are described in Table 1. The Brunet-Lézine Scale showed that the hand-eye coordination was the area in which children presented with most delays. According to the Bayley III Scale, the gross motor area was the one presenting more delays, however, the mean was within expectations. As to the analyses of concurrent validity, no correlation was found between DQP and gross motor skills (ρ=0.304; *p*=0.090), nor between the DQS and the socioemotional score (ρ=0.234; *p*=0.198). There was moderate correlation between the hand-eye coordination and the fine motor score (ρ=0.448; *p*=0.01), and very weak correlation between DQL and the language score (ρ=0.383; *p*=0.030).

**Table 1:**
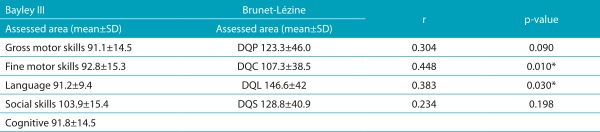
Correlation between the Bayley III Scale and the Brunet-Lézine Scale per area for Group 1 (32 patients aged 1 to 6 months old).

SD: Standard deviation. Corrected Bayley III Scale Scores (-7): developmental quotient for posture; DQC: quotient for hand-eye coordination; DQL: developmental quotient for language; DQS: developmental quotient for social reactions.

Table 2 presents the performance of children and the correlation per area between the scales applied in Group 2. According to the Brunet-Lézine Scale, in Group 2, a child presented with delay in language development. Considering the Bayley Scale, children in Group 2 performed worse in the gross motor field, being 5 of them (13.9%) classified with developmental delay. By making analyses of correlation between the scales, moderate correlation was found between DQP and gross motor skills (ρ=0.484; *p*=0.003), between hand-eye coordination and fine motor score (r=0.489; *p*=0.002), and between DQS and socioemotional score (r=0.435; *p*=0.008). However, no correlation between the variables was found for the language area (ρ=0.252; *p*=0.138).

**Table 2:**
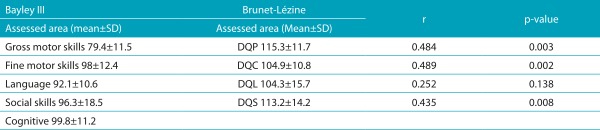
Correlation between the Bayley III Scale and the Brunet-Lézine Scale per area for Group 2 (36 patients aged from 6 to 12 months old).

SD: Standard deviation. Corrected Bayley III Scale Scores (-7): developmental quotient for posture; DQC: quotient for hand-eye coordination; DQL: developmental quotient for language; DQS: developmental quotient for social reactions.

Table 3 shows the performance of Group 3 in each scale, and the correlation values. Considering the Brunet-Lézine Scale, the children presented with lower mean in the sociability area, and four of them manifested delay in the language area. Likewise, when assessed by the Bayley III Scale, the children presented the expected performance in the field of language, also with four children presenting delay in social skills. When both scales were correlated, it was possible to verify that there was moderate correlation between DQP and gross motor score (ρ=0.468; *p*=0.037), and a very strong correlation between DQL and the language score (r=0.890; *p*<0.001). However, no correlation was found between DQC and fine motor score (r=0.385; *p*=0.094), and between DQS and socioemotional score (r=0.225; *p*=0.340).

**Table 3:**
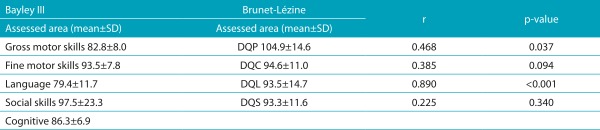
Correlation between the Bayley III Scale and the Brunet-Lézine Scale domains per area for Group 3 (20 patients aged from 18 to 24 months old).

SD: Standard deviation. Corrected Bayley III Scale Scores (-7): developmental quotient for posture; DQC: quotient for hand-eye coordination; DQL: developmental quotient for language; DQS: developmental quotient for social reactions.

The percentage rates of sensitivity, specificity, positive predictive value (PPV), negative predictive value (NPV), and accuracy of the Brunet-Lézine Scale are presented in Table 4. As to sensitivity, the Brunet-Lézine scale presented, in all areas, values that were below expectations, as it was hardly sensitive in the gross motor and sociability areas, and little sensitive in the fine motor area. The specificity analyses in the Brunet-Lézine Scale indicated high specificity in all developmental areas. This scale presented low PPV in all assessed areas; NPV in this scale was acceptable for gross motor skills, good for sociability, and high for the fields of language and fine motor skills. The diagnostic percentage of the Brunet-Lézine Scale, compared to the Bayley III Scale, was lower in the fields of sociability and gross motor skills, similar in the field of fine motor skills and higher in the language area.

**Table 4:**
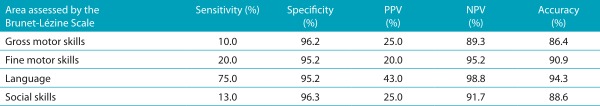
Sensitivity, specificity, positive and negative predictive value, and accuracy of the Brunet-Lézine Scale.

% percentage; PPV: positive predictive value; NPV: negative predictive value.

## DISCUSSION

Up until now, the literature did not show studies assessing the concurrent validity of any versions of the Brunet-Lézine Scale [version of the authors, modified version, and revised version (BLR)].^10^ ^,^ ^11^ In this sense, the Bayley III Scale was used to assess this psychometric data.

Considering the gross motor skills, moderate correlation was found between both scales in Groups 2 and 3, values of 0.484 and 0.468, respectively. The same correlation (moderate; 0.54) was found between gross motor score (Bayley III Scale) and the Psychomotor Development Index (PDI), in Bayley II Scale, among 57 children aged 1-42 months. Between the Bayley III Scale and the Peabody Developmental Motor Scale - Second Edition (PMDS-2), there was similar moderate correlation (0.59) between the gross motor score (Bayley III Scale), and the gross motor quotient (PMDS-2) of 81 children aged 2 to 42 months.^21^

In the fine motor skill area, there was positive moderate correlation between the modified version of the Brunet-Lézine Scale and the Bayley III Scale in Groups 1 (0.45) and 2 (0.49). In another study, it was possible to observe that the correlation of the fine motor score in the Bayley III Scale, with the PDI of the Bayley II Scale, was moderate (0.52). In another study conducted by the authors of the Bayley Scale, the fine motor score (Bayley III Scale) was compared with the motor fine quotient and with the visual-motor integration subtest, both in the PDMS-2, in which both correlations were moderate (0.59 and 0.55, respectively).^21^ In this study, the fine motor correlations in Groups 1 and 2 were moderate, as well as the correlation between the Bayley III Scale and other scales assessing fine motor skills.

In the field of language, there was very weak positive correlation between DQL (Brunet-Lézine) and the score composed of language (Bayley III Scale) in Group 1 (0.383), and there was strong positive correlation between both scores in Group 3 (r=0.890). By correlating the composed language score (Bayley III Scale) with the Mental Developmental Index (MDI) in the Bayley II Scale, as evident in the study published by the scale manual, there was strong correlation between them (0.71). A recent study that analyzed the presence of correlation between the language area of these two scales, indicated that there was strong correlation (0.81) between the language score in the Bayley III Scale and the MDI score.^18^ When assessing the Bayley III Scale and another scale specific for language (Preschool Language Scale Fourth Edition - PLS-4), there was moderate correlation (0.66) between the composed scores, assessed in 69 children aged 5-42 months.^21^ It is worth mentioning that the correlation with higher value between the scales analyzed here was verified in the language area of Group 3.

By observing the social skills, there was moderate correlation in Group 2, higher than the one found in the specific analysis of the scale, which compared the socioemotional scores of the third version of the Bayley Scale, mental (MDI) and behavioral classification (Behavior Evaluation Scale for Children - BRS). In both correlations, between the children mentioned in the study presented in the Bayley Scale, the level of strength was weak, with values ranging from 0.25 to 0.37, respectively.^21^ Values that low can be explained by the lack of scales that assess only socioemotional matters, as was the case of the previous version of the Bayley Scale.

When both test formats are used for important decision-making, the correlations must be very high, around 0.95.^8^ Considering this statement, it is not possible to ensure that the Brunet-Lézine Scale is efficient to assess the neuro psychomotor development of children aged 1 to 24 months. However, by considering that strong correlations are a good level of relationship between the tests, it is observed that the Brunet-Lézine was more appropriate to assess the language of children aged between 18 and 24 months. In the other variables, when correlation was present, it was mostly moderate, which does not show the effectiveness of the evaluation of the area, since, in this study, the Bayley III Scale was considered as gold standard.

Sensitivity measures the ability of a test to properly detect individuals with a specific disease or condition.^5^ ^,^ ^9^ Based on that, high values are essential for a discriminatory test to be classified as good, because children with developmental delay should not be left without a diagnosis. The earlier the intervention begins, the better the prognosis.^3^ The modified version of the Brunet-Lézine Scale presented low sensitivity in all areas, ranging from 10% in the fine motor area (weak), to 75% in language (regular), which requires attention from the evaluators, adding to the evaluation a full observation of the children’s development. The Brunet-Lézine Scale showed high values of specificity in all areas of development, that is, it identifies children without delay. The percentages of specificity ranged between 96.3% in social skills, and 95.2% in fine motor and language areas, all with specificity rates above recommendation.^4^ As to the PPV, the Brunet-Lézine Scale showed low numbers in all areas, ranging between 20% for fine motor skills and 43% for language. This means that a few individuals who had actual delay were among the diagnosed ones. On the other hand, this scale showed good NPV, ranging between 89.3%, in gross motor skills, and 98.8% in language. In the language area, the Brunet-Lézine Scale had high ability to identify individuals without any alteration. It is worth mentioning that predictive values are very much influenced by the prevalence of the event in the analyzed population, and probably did not reflect completely the reality of this scale as to the predicted development. Finally, the Brunet-Lézine Scale presented acceptable accuracy percentage rates in this study, ranging between 86.4% in gross motor skills, and 94.2% in language. Therefore, its ability to conduct proper diagnoses, both positive and negative, was not strong.

The choice of the researchers to use the Bayley III Scale as gold standard to assess the development of children, aimed at identifying more delays than its previous version, because, according to the Flynn effect,^24^ scales created recently should be more strict as to the analysis of development. However, this characteristic was not observed in other studies conducted with the Bayley III Scale.^13^ ^,^ ^16^ ^,^ ^18^ ^,^ ^21^ ^,^ ^25^ ^,^ ^26^ ^,^ ^27^ ^,^ ^28^ ^,^ ^29^ ^,^ ^30^Because of that, it was necessary to conduct a 7-point correction of the scores in the Bayley III Scale.

On the basis of the results, it is possible to observe that the Brunet-Lézine Scale presented strong correlation as to the language in Group 3. This suggests that it is valid to assess children aged between 18 and 24 months in the language area. The Brunet-Lézine Scale showed low sensitivity and high specificity in all areas, besides PPVs below recommendation, and NPVs above recommendation in all areas. Accuracy was within the acceptable limits. It is worth mentioning that these analyses may have been damaged by the low prevalence of delay in this sample.

After all the analyses in this study, it was possible to notice that both scales have limitations, corroborating literature, which supports the idea that there are no perfect scales to assess development among children.^4^ ^,^ ^5^ However, it is important to prioritize scales that are easy to apply, with good cost-benefit, and good psychometric qualities to help the clinical judgment of the professional involved.^4^ ^,^ ^5^ ^,^ ^8^ ^,^ ^12^ ^,^ ^26^ The current study reinforces the clear need of health professionals and researchers to use valid and reliable instruments to assess the development of children. In this sense, in case it is not possible to use the Bayley III scale in some clinical situations, the Brunet-Lézine scale can be a useful tool in the follow-up of preterm children in the follow-up of outpatient clinics.

This study provides clinical information to several health professionals who follow-up preterm children, with low birth weight, concerning the use of standardized diagnostic methods that are sensitive to identification, and propose an intervention in cases of psychomotor developmental delay.
